# Single Digit Index Finger Amputation—To Replant or Not?

**DOI:** 10.1177/22925503211024753

**Published:** 2021-08-18

**Authors:** Marshall Thibedeau, Maleka Ramji, Madeleine McKenzie, Justin Yeung, Duncan A. Nickerson

**Affiliations:** 1 Cumming School of Medicine, University of Calgary, Alberta, Canada; 2 Section of Plastic Surgery, University of Calgary, Alberta, Canada; 3 Department of Biology, Concordia University, Montreal, Quebec, Canada

**Keywords:** amputation, traumatic, Canada, finger injuries, replantation, revision amputation, surveys and questionnaires

## Abstract

**Background:** Single index finger replantation is often listed as a contraindication due to its hindrance of hand function when replanted. Recent studies demonstrate comparable subjective and global functional outcomes for index flexor zone II finger replants versus revision amputations. We therefore sought to identify current opinions of plastic surgery trainees and staff treating single index finger zone II amputations including influential patient and injury characteristics. **Methods:** With the approval of the Canadian Society of Plastic Surgery, a 17-question survey was sent via email to all listed members on 3 separate occasions. Participation was voluntary and survey responses were compiled and analyzed using SPSS statistical software. **Results:** Survey response rate was 38.5%. When asked whether the surgeon would replant a single index digit, flexor zone II, sharp amputation, 55.3% of respondents chose “yes,” while 44.7% responded “no.” Staff (51.5%) were less likely to replant a single index digit amputation. Likelihood of replant dropped substantially in crush (12.4%) and avulsion (17.1%) injury. Smoking was the most likely patient characteristic to change a surgeon’s decision (61.9%). Poor range of motion (77.5%) and patient satisfaction (72.5%) were the most frequently listed reasons not to replant. **Conclusion:** Among Canadian plastic surgeons, there exists disagreement in how single index flexor zone II amputations should be managed. In review of the literature, these notions and previous teaching around replants highlight many inherent surgeon biases with regard to the merit and value of single digit replantation.

## Abstract (fr)

**Contexte:** La réimplantation isolée de l’index est souvent considérée comme une contre-indication compte tenu de la gêne causée au fonctionnement de la main une fois réimplanté. Des études récentes démontrent des résultats fonctionnels, subjectifs et globaux, comparables pour les réimplantations digitales du fléchisseur de l’index en zone II comparativement aux amputations de révision. Nous avons donc cherché à identifier l’avis actuel des stagiaires et du personnel de chirurgie plastique traitant des amputations isolées en zone II, incluant les caractéristiques de la blessure et l’influence des patients. **Méthodes:** Une enquête comportant dix-sept questions a été envoyée à trois reprises avec l’approbation de la Société canadienne de chirurgie plastique aux adresses de courriel de tous les membres listés. La participation était volontaire et les réponses à l’enquête ont été compilées et analysées au moyen du logiciel statistique SPSS. **Résultats:** Le taux de réponse à l’enquête a été de 38,5 %. À la question de savoir si le chirurgien réimplanterait un index isolé, fléchisseur zone II, après amputation nette, 55,3 % des répondants ont choisi « oui » et 44,7 % ont répondu « non ». Le personnel (51,5 %) était moins susceptible de réimplanter un index après amputation isolée. La probabilité de réimplantation diminuait nettement en cas de lésion par écrasement (12,4 %) et arrachement (17,1 %). Le tabagisme était la caractéristique du patient le plus susceptible de modifier la décision d’un chirurgien (61,9 %). Une mauvaise amplitude de mouvement (77,5 %) et satisfaction du patient (72,5 %) ont été les raisons citées le plus souvent pour ne pas réimplanter. **Conclusion:** Il existe, parmi les chirurgiens plasticiens canadiens, un désaccord sur la manière dont les amputations isolées d’un index (fléchisseur zone II) doivent être gérées. La revue des publications montre que ces notions et l’enseignement antérieur concernant les réimplantations soulignent les multiples biais inhérents aux chirurgiens pour ce qui concerne l’intérêt et la valeur de la réimplantation d’un seul doigt.

## Introduction

Traumatic amputations of the digits are common and occur both in the workplace and at home.^1-3^ Management of such injuries require decision-making between revision amputation or digit replantation. This is often a difficult decision for both the patient and the surgeon, and a myriad of factors must be contemplated: feasibility of replant, functional outcomes specific to the biomechanics of the injured finger, implications for the patient’s daily activities, work status, and estimated time for recovery.

While digit replant survival has improved over the decades, functional outcomes of flexor zone II injuries have historically been poor, typified by tendon adhesions, bony non-union, and poor sensory return.^4-8^ A study by White “Why I hate the Index finger,” published in 1980, highlights the limitations specifically of index replants.^5^ In it, he entertainingly expresses, “After sustaining an inconsequential injury as a result of its own arrogance, it will refuse to perform regardless of circumstances. Not only does it refuse to function, it interferes with the uninjured parts of the hand engaging in useful activity. It does this by standing in the way, fully extended, inviting disaster.” His manuscript continues to inform the attitude towards the index as an especially poor candidate for replantation.^9-11^ This has led to considerable animus towards single digit flexor zone II replants with contemporary references listing it as a relative contraindication^12-16^ particularly in the index.^5-7^ Even more, an esteemed resource for hand surgery education, Green’s operative Hand Surgery continues to list individual finger amputations in an adult at a level proximal to the flexor digitorum superficialis (FDS) insertion (particularly in the index or small fingers) as a contraindication to replant.^17^ Due to this, single index finger replantation is often avoided and if replanted, viewed as a hindrance to ultimate hand function.^6,9,11,18^

Recent studies, however, demonstrate comparable subjective and global functional outcomes for flexor zone II digit replants when compared to revision amputation.^19,20^ Considering this, we sought to identify if the historic bias against replant of the index finger zone II persists, or if preference is made for replantation. Furthermore, we were curious to learn what specific injury, patient, or training characteristics might sway surgeons towards replantation versus revision amputation of these injuries.

## Methods

Ethics approval (REB19-0180) was provided by the University of Calgary’s Conjoint Health Research Ethics Board. After obtaining ethics approval, a 17-question survey assessing whether plastic surgeons would replant a single index finger, zone II amputation was created (see Supplementary Appendix I). Questions relating to which injury and patient characteristics might influence a surgeon to replant versus perform revision amputation, along with respondent demographics, were included. Questions regarding why a surgeon would choose not to replant a single index digit were also included. Each question had an open script section for participants to add additional information to provide rationale their answers. The survey was created using a cloud-based software that allows for the development and distribution and data analysis of customizable surveys.

With the approval of the Canadian Plastic Surgery Society (CSPS), the survey was sent via email to 593 members. Participation was voluntary and the survey was distributed on 3 separate occasions to maximize response rate. The final survey was sent in October 2019. Survey responses were compiled and analyzed using SPSS statistical software. Chi-square tests were performed for demographic subgroup analysis using Microsoft Excel.

## Results

A total of 244 surveys were returned. Of those, 228 (93%) of the surveys were considered complete yielding a response rate of 38.5%. These were evaluated for data extraction and analysis. Survey respondents included attendings (69.7%), fellows (3.5%), residents (14.9%), and an additional 11.8% of respondents who didn’t identify their designation. The majority of respondents were from Ontario (32.9%), while minority of responses came from Manitoba (2.6%), Saskatchewan (2.6%), and New Brunswick (1.8%). With regard to practice type, 59.7% of respondents work in academic centres, while 28.5% identify working at a community hospital. When identifying number of practice years, majority of responders have greater than 20 years of practice (22.8%), followed by less than 5 years of practice (18.9%); 34.2% and 31.1% of respondents have completed a hand and upper extremity surgery or microsurgery fellowship, respectively. Demographic data are summarized in Table 1.

**Table 1.** Demographics of Survey Respondents.

Category	Number (%)^a^	Category	Number (%)
Level of training		Fellowship training	
Resident	34 (14.91)	Hand and upper extremity	78 (34.21)
Fellow	8 (3.51)	Microsurgery	71 (31.14)
Attending	159 (69.74)		
Demographic not provided	27 (11.84)		
Province of practice		Estimated single digit replants per year	
British Columbia	38 (16.67)	0	95 (41.67)
Alberta	44 (19.30)	1	37 (16.23)
Saskatchewan	6 (2.63)	2	26 (11.40)
Manitoba	6 (2.63)	3	24 (10.53)
Ontario	75 (32.90)	4	6 (2.63)
Quebec	19 (8.33)	5	3 (1.32)
New Brunswick	4 (1.75)	>5	10 (4.39)
Prince Edward Island	0 (0)		
Nova Scotia	9 (3.95)	Provide on call services	
Newfoundland	0 (0)	Yes	191 (83.77)
		No	10 (4.39)
Years of experience			
0	23 (10.09)	Primary site of practice	
<5	43 (18.86)	Academic	136 (59.65)
5-10	28 (12.28)	Community	65 (28.51)
10-15	33 (14.47)		
16-20	22 (9.65)	Total complete responses	228
>20	52 (22.81)	Total complete response with demographics	201 (88.16)

^a^ Twenty-seven surgeons completed the survey but did not provided demographic details. Percentages are based on those who completed the survey regardless of whether demographic data were provided (228).

When asked whether the surgeon would replant a single index digit, flexor zone II, sharp amputation, 55.3% of respondents chose “yes.” Conversely, 44.7% of respondents said they would not replant a single index digit, zone II sharp amputation. Those who responded with “no” did not answer any further survey questions with regard to replantation of a single index digit index. Survey response by demographics is summarized in Table 2.

**Table 2.** Demographics of Survey Responses.

Category	Yes—number (%)	No—number (%)
Level of training		
Resident	22 (64.7)	12 (35.3)
Fellow	2 (25.0)	6 (75.0)
Attending	79 (49.7)	80 (50.3)
Undefined	23 (85.2)	4 (14.8)
Total	126 (55.3)	102 (44.7)
Fellowship training^a^		
Hand and upper extremity	32 (43.8)	41 (56.2)
Microsurgery	38 (55.9)	30 (44.1)

^a^ Attendings with completed fellowship training.

When matching decision to replant with demographic data, staff (51.5%) were less likely to replant a single index digit amputation, when compared to residents (35.3%). Surgeons with greater than 10 years of experience were less likely (44.9%) to replant than those surgeons with less than 10 years of experience (58.5%). Furthermore, those with a hand and upper extremity surgery fellowship were less likely to replant a single digit amputation (43.8%) versus those with a microsurgery fellowship (55.9%).

Only 52.7% of respondents had replanted a single finger amputation within the last year. The majority of whom had only performed 2 or less of these operations within the last year (59.4%). Surgeons who had replanted a single finger amputation within the last year chose to replant 55.7% of the time compared to 46.3% of those who had not performed a replant within the last year.

**Figure 1.**
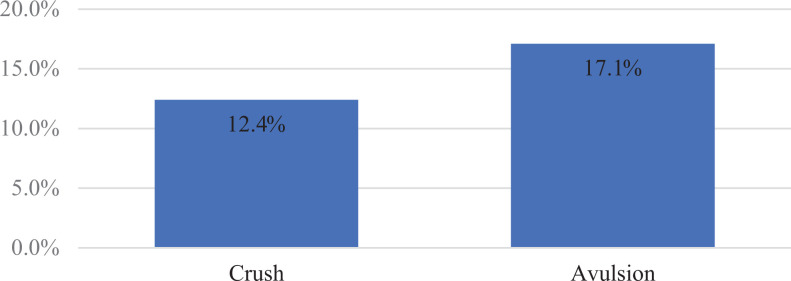
Change of mechanism: opted to replant.

Of those who would replant, when the mechanism was changed to avulsion or crush, the likelihood of replant decreased substantially. Here, 82.9% and 87.6% opted to not replant a single index finger, zone II amputation for avulsion or crush injury, respectively (Figure 1). Surgeons qualified their responses by explaining that each digit would need a bedside or intraoperative assessment, with exploration of vessels.

**Figure 2.**
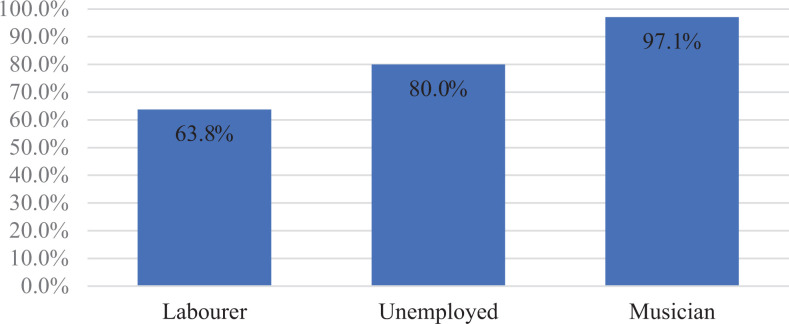
Profession: opted to replant.

Considering patient’s occupation, surgeons were more likely to replant the digit if the patient was a musician, replanting 97.1% of cases. Decision to replant was less likely, if the patient was unemployed (80.0%) or a labourer (63.8%; Figure 2).

**Figure 3.**
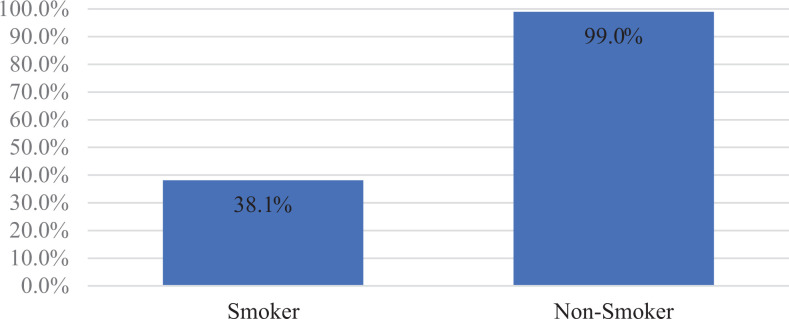
Smoker: opted to replant.

When accounting for smoking status, surgeons would replant 99% of time in non-smokers but this was reduced to only 38.1% of the time in smokers. Few surgeons qualified their response by noting that if the patient was to quit smoking post-operatively, they would replant even if the patient was a smoker at time of injury. Decision to replant was also dependant for some, based on patient pack per year history and on smoking-related medical comorbidities (Figure 3).

When considering patient age, approximately one-third of surgeons (34.6%) would replant a single digit amputation, regardless of age; 58.7% of surgeons would perform replantation for patients only if they were younger than 65 years of age.

**Figure 4.**
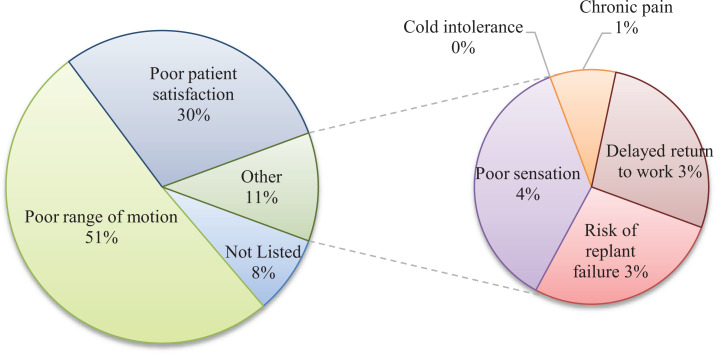
Primary reason not to replant.

Of the 44.7% of respondents who opted not to replant a single digit, index finger, zone II amputation, poor range of motion (51.0%) and poor patient satisfaction (29.6%) were by far the most frequent reasons for foregoing replant (Figure 4).

**Figure 5.**
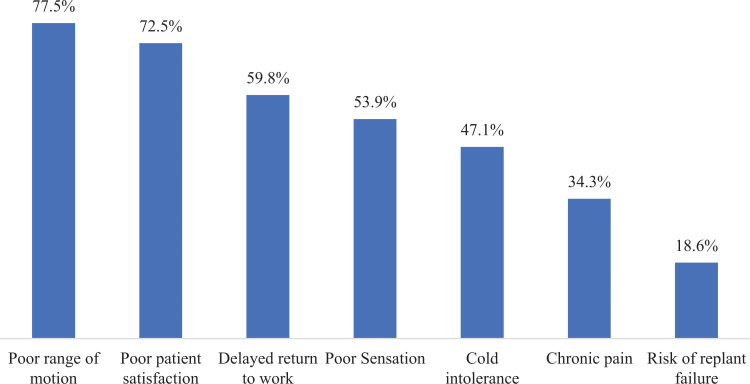
All reasons not to replant, % respondents.

When those surgeons who opted not to replant and were allowed to select all factors that influenced this decision, poor range of motion (77.5%), poor patient satisfaction (72.5%), and delayed return to work (RTW; 59.8%) were the 3 most frequently selected. Poor sensation (53.9%) also figured prominently as a reason to not replant (Figure 5).

## Discussion

The completed survey response rate was 38.5%, after 3 distributions from April to October 2019, which is comparable to expected online survey physician specialist response.^21^ Response representativeness is another measure to be considered, which was determined by Cook et al to be an additionally important element of survey research.^22^ With consideration of level of training, the survey captured 25.4% (159 of 627 practicing plastic surgeons listed by Canadian Medical Association (CMA)) and 28.8% (34 of 118 residents enrolled by the Canadian Resident Match Service between 2015 and 2019), respectively. This is confounded by 27 respondents who completed the survey but did not complete the demographics section. In addition, 85.3% of resident responses were from Alberta, Ontario, and Quebec; and survey responses may therefore disproportionally represent the respective training bias and practice tendencies within these provinces. In terms of regional representation, a notable deficit is the total response rate from Quebec at 8.3% (19/228) despite comprising 24% of practicing plastic surgeons. Although the survey was distributed to Quebec plastic surgeons who were included in the CSPS members mailing list, it was not translated in French, which may account for the lack in this group’s participation.

The primary scenario involved deciding on whether to replant a dominant hand flexor zone II index amputation from sharp/guillotine mechanism. This scenario was designed intentionally to emphasize the potential hindrance of activities of daily living (ADLs) and employment (dominant hand involvement) and to minimize survivability and mechanism considerations (sharp mechanism of injury) in an amputation commonly associated with poor functional outcomes (index and flexor zone II). Despite historical review articles and summary evidence listing this type of amputation as a relative contraindication for replant, the survey results demonstrated a clear non-consensus among respondents on whether to replant versus not to replant (55.3% vs 44.7%).^5,11,14-16^

Increased surgical experience had an impact on the likelihood to proceed with revision amputation; 51.5% of attendings opted not to replant while fewer residents (35.3%) favoured not replanting. Among attendings, those with greater than 10 years of experience opted to replant 44.9% of time versus 58.5% for those with fewer than 10 years of experience. Furthermore, a larger percentage of hand and upper extremity fellowship trained surgeons (56.2%) opted not to replant a single digit index amputation, when compared to microsurgery fellowship trained surgeons (44.1%). This finding may reflect individual surgeon’s hierarchical considerations for a successful procedure. A hand surgeon may weigh functional outcomes of range of motion and sensation, above digit viability. By contrast, a microsurgeon may opt to replant a digit, satisfied with its initial viability, only to weigh the functional outcomes later in the patient’s post-operative journey. Additionally, hand fellowship trained surgeons are often the ones who perform secondary procedures for these single digit replants and therefore may simply have more insight and exposure into the functional hindrance a single digit replant can have on overall hand function. In the free form comments, surgeons cited the importance of a shared decision-making model with the patient, emphasizing discussions around prolonged rehabilitation and time off work (TOW). Psychosocial considerations such as female sex and the patient’s ethnic and cultural background were also mentioned by surgeons as factors to consider in decision-making.

Fifty-two percent of the surgeons had performed a single finger replant within the last year with the majority having performed only 1 or 2 of these operations (59.4%). The surgeons were predominately from academic institutions (59.7%). This highlights the infrequent nature of the single finger replantation. Moreover, attributing experience significant enough to alter decision-making in those who had performed 1 replant as compared to no replants does not pass muster. In particular, as in this survey, when there is neither standardized nor agreed upon specific criteria on when surgery should be pursued. Concentrating replant cases to major centres where transport is within acceptable ischemic times could bolster surgeon experience and ultimately patient outcomes.

A recent study conducted by Zhu et al involving 1023 patients with traumatic single digit amputations supports those respondents who opted to replant. They stratified level of injury by the Tamai classification, with combined Tamai Levels IV and V corresponding with the region of flexor zone II injuries. The replants were assessed using the Michigan Hand Outcomes Questionnaire (MHQ), which involves an average score from 0 to 100, with higher scores indicating better results in overall hand function, work performance, ADLs, pain, aesthetics, and patient satisfaction. The MHQ scores for index level flexor zone II replants were higher and statistically significant compared to revision amputations at the same level. Replant patients, however, were hospitalized 8 days longer, required longer sick leave (12 vs 3 weeks), and cost more on average compared to revision amputations.^19^ This study provides compelling evidence to consider single index flexor zone II replants, which contrasts with White’s assertion and continued teaching around flexor zone II amputations as a contraindication for replantation.

Waikakul et al found that extensive crush and avulsion amputations of single digits resulted in the poorest global assessment of functionality using Chen criteria. This supports findings from our survey, where surgeons who were willing to replant single digit amputations were much less inclined in the setting of avulsion and crush. Vessel integrity and digit viability are commonly assessed to determine likelihood of successful replant. A surgeon should equally weigh the potential final functional outcomes, such as nerve recovery and anticipated range of motion when deciding to pursue replant or not.^23^

Of patient characteristics, smoking status was the most likely “yes scenario” modifier selected by surgeons to opt not to replant (61.9%). The literature, however, has considerable variability around the impact of smoking on digit survival in replants. Waikakul et al compared replant survival based on an active smoking habit and found a significant impact on digit survival (61.1% vs 96.7% for non-smokers),^23^ with other articles making similar conclusions.^24,25^ By contrast, recent studies have found no statistically significant increased risk of replant failure in smokers.^26-28^ These conflicting findings can be clarified by considering smoking in a dose-response fashion as described by Zhu et al in 2017. By stratifying smokers by cigarettes per day, they found that only smokers with a habit of >20 per day cigarettes had an increased risk of replant failure (76.2%) when compared to survival for non-smokers (92.4%).^29^

Of those surgeons who were initially in favour of replanting, approximately one-third (27.9%) set an upper age limit of 65 years or less as the threshold to perform replantation. Digit amputations of any type are generally indicated for children despite poorer replant survival rates.^11,28,30-33^ This is on account of the favourable functional outcomes and high patient satisfaction following rehabilitation of the replanted digit.^23,34-37^ Advanced age has been associated with increased risk of replant failure^29^ particularly in ages >70 years^38^ with reported survival rates of 70% to 87%. One large scale study suggests that the higher failure rate in elderly populations is indicative of the higher prevalence of comorbidities associated with replant failure which when controlled for shows no statistically significant risk of replant failure with increasing age.^39,40^ When controlling for mechanism of injury by age distribution, age was a statistically significant factor impacting functional outcomes, specifically poor sensory return.^41^ Patient satisfaction, however, remained high at 94% despite poorer functional outcomes.^38^ Collectively, this suggests that the decision to replant in the elderly population should focus primarily on relevant medical comorbidities and the impact of prolonged and extensive rehabilitation on quality of life.

Based on the patient’s occupation, surgeons were more likely to replant a single digit if the patient was a musician (97.1%) and least likely if they were a labourer (63.8%). Those who provided comments on this decision rationale emphasized the value of a shared decision-making model irrespective of profession, while few focused on heavy labourers and their early RTW as a meaningful consideration. Objectively, post-operative outcomes and digit viability do not change with profession, and yet according to this survey, surgeons are one-third of the time less likely to attempt single digit replant in a manual labourer. The decision to forego replant has long been explained by the desire for early RTW and avoidance of rehabilitation in the manual labour population. Interestingly, large scale meta-analysis by Harris et al did find a strong association between compensation status for workplace injury and poor subjective outcome after surgery.^42^ Studies evaluating objective measures have likewise demonstrated reduced hand function and delayed RTW, when unsettled worker’s compensation claims or ongoing litigation was present.^43,44^

In the cohort of surgeons who opted not to replant a single index flexor zone II amputation, the 3 most frequent factors that influenced this decision included poor range of motion (77.5%), poor patient satisfaction (72.5%), and delayed RTW (59.8%).

Poor patient satisfaction is a unique modifier for decision- making, as it is a subjective measure and varies considerably from patient to patient. Patient reported outcomes today are considered among the gold standard for post-operative evaluation and its application is aided by the prevalence of standardized questionnaires such as the disability of the arm, shoulder, and hand score (DASH). A study by El-Diwany et al looked specifically at replantation versus primary revision amputation in flexor zone II amputation of any digit. They found no statistically significant difference between the cohorts’ DASH or Beck Depression Inventory scores. Further, when asked, a higher percentage of patients in the replantation group would opt for repeat replant than revised patients would opt again for revision amputation.^20^

When index amputations are treated with ray amputation as opposed to revision amputation at the level of injury, Melikyan et al found index ray amputations had an increased DASH score compared to other fingers.^45^ The index cohort, however, had a higher rate of traumatic amputations which has been demonstrated to negatively impact DASH scores.^46,47^ In the event that a failed replant undergoes secondary ray revision, there appears to be no significant difference in DASH scores.^48^ Fortunately, when it comes to subjective outcomes, there appears to be a component of time-dependant functional adaptation with those >3 years since injury reporting an average DASH 30% lower than the <3-year cohort.^49^

Interestingly, delayed RTW was listed as the primary reason not to replant by only 3.1% of respondents but was the third most frequent multifactorial consideration (59.8%). A 2015 study by El-Diwany et al found no statistical significance of sick days used or rate of return to the same occupation between replantation and revision amputation groups in flexor zone II injuries with a higher prevalence of worker’s compensation patients in the revision cohort (16.5 vs 15.3 weeks).^20^ This is in contrast to the study of Zhu et al (2018) that found replants to require on average 12 versus 3 weeks off for revision amputations. Replants in this study required less sick days than revision amputations in the study by El-Diwany. Confounders that may account for this could include the occupational demographics, societal supports, and the labour regulatory differences between Canada and China.^19^ In addition, Bhat et al specifically looked at secondary revision after failed replantation versus primary ray amputation and likewise found no significant differences in TOW (15.5 vs 13 weeks) despite the requirement for an additional surgery.^48^ One counterpoint to these studies is seen in a study by Peimer et al, which found an average TOW of 9 weeks in primary ray revision versus 16 weeks in secondary ray revision.^44^ Neither one of the latter 2 studies compared successful replants to their revision groups and therefore should be used cautiously for their RTW conclusions.

A primary limitation of the study is response bias, given its survey design and reflected in the response rate of 38.5%. Our survey invites were sent to the mailing list of a professional association and participation was voluntary. The results of the subgroup analysis (staff vs residents, fellowship influence on decision-making, and replant performed in the last year) were not statistically significant. The lack of statistical significance among the subgroups demonstrates a clear non-consensus on surgical indications for single digit replantation. This highlights the prevalence of individual surgeon bias in decision-making. Further, given the survey design of optional commentary for the multiple choice questions, surgeon rationale for their choices was elicited but not provided in great quantity to make meaningful conclusions. Inclusion of residents, who as a group favoured replantation (65%), may have skewed the results. Residents typically lack long-term follow-up with their surgical cases and have a training incentive to pursue more technically challenging operations.

## Conclusion

Traumatic single digit amputations are devastating injuries, and replantation is a time-sensitive salvage treatment. Among Canadian plastic surgeons, there exists disagreement in how single index flexor zone II amputations should be managed between replant or revision amputation. In review of the literature, these notions and previous teaching around replants highlight many inherent surgeon biases with regard to the merit and value of single digit replantation. Review of current replant indications should be revised and standardized to consider level of injury and mechanism guided by anticipated global outcomes.

## Supplemental Material

Supplemental Material, sj-docx-1-psg-10.1177_22925503211024753 - Single Digit Index Finger Amputation—To Replant or Not? Click here for additional data file. Supplemental Material, sj-docx-1-psg-10.1177_22925503211024753 for Single Digit Index Finger Amputation—To Replant or Not? by Marshall Thibedeau, Maleka Ramji, Madeleine McKenzie, Justin Yeung and Duncan Alexander Nickerson in Plastic Surgery
