# Supplementary material for: Single Digit Index Finger Amputation—To Replant or Not?
Source: Plast Surg (Oakv). 2021 Aug 18;31(1):44–52. doi: 10.1177/22925503211024753 (PMC9900044; doi:10.1177/22925503211024753)
Supplement: Supplemental Material, sj-docx-1-psg-10.1177_22925503211024753 - Single Digit Index Finger Amputation—To Replant or Not? [file sj-docx-1-psg-10.1177_22925503211024753.docx]

| **Survey Contents: Single Finger Amputation – to Replant or Not?** | | |
| --- | --- | --- |
| Section | Question | Possible Answers |
| **Study Description**   - Staff Authors and affiliated university - Study Purpose - Ethics approval with statement of risks - Estimated time and number of survey question - Anonymity of responses and voluntary participation | Do you consent to participate? | 1. Yes 2. No (terminated survey) |
|  | | |
| **Primary Decision**: Please read the following cases presented below. With the provided information, please answer yes or no to the clinical question. | An adult patient amputates their dominant hand’s index finger at the flexor zone II level. Would you  replant if the mechanism of injury was sharp/guillotine? | 1. Yes (Participant sent to **clinical scenario A**) 2. No (Participant sent to **clinical scenario B**) 3. Comment Box (optional) |
|  | | |
| **Clinical Scenario A**: Please read the following cases presented below. With the provided information, indicate scenarios where you would recommend to replant the digit. All injuries involve a **single digit, index finger amputation, in flexor level zone II**. | What is the upper limit in age, where you would suggest replantation? (if there is no max age, please write 99) | Open comment box |
|  | Would you replant, in light of their smoking status? | 1. Smoker, yes or no 2. Non-smoker, yes or no 3. Comment box (optional) |
|  | Would you replant, in light of the following mechanism? | 1. Crush, yes or no 2. Avulsion, yes or no 3. Comment box (optional) |
|  | Would you replant, in light of their occupation? | 1. Unemployed, yes or no 2. Labourer, yes or no 3. Musician, yes or no 4. Comment box (optional) |
|  | Please provide any relevant comments | Open comment box |
|  | | |
| **Clinical Scenario B** | What anticipated outcome most influenced your decision not to replant? | (Choose only one answer)   1. Risk of replant failure 2. Poor range of motion 3. Poor Sensation 4. Cold Intolerance 5. Chronic pain 6. Poor patient satisfaction 7. Delayed return to work 8. Other (please specify) |
|  | What anticipated outcomes influenced your decision not to replant? | (Choose all that apply)   1. Risk of replant failure 2. Poor range of motion 3. Poor Sensation 4. Cold Intolerance 5. Chronic pain 6. Poor patient satisfaction 7. Delayed return to work |
|  | Please provide any relevant comments. | Open comment box |
|  | | |
| **Participant Demographics** | What is your level of training? | Resident, Fellow, Staff |
|  | How many years have you been in practice? | 0, < 5, 5 – 10, 10 – 15, 16 - 20, >2 0 years |
|  | Have you done a fellowship in hand or microsurgery? | (Choose all that apply)   1. Yes – Hand and Upper Extremity Fellowship 2. Yes – Microsurgery Fellowship 3. No |
|  | What is your primary site of practice? | 1. Academic 2. Community |
|  | In what province do you practice? | List of provinces |
|  | Do you provide on call emergency hand surgery services? | 1. Yes 2. No |
|  | What is the estimated number of single finger replants you have performed in the last year? | None, 1, 2, 3, 4, 5, > 5 |
